# Tissue-specific tagging of endogenous loci in *Drosophila melanogaster*

**DOI:** 10.1242/bio.016089

**Published:** 2015-12-23

**Authors:** Kate Koles, Anna R. Yeh, Avital A. Rodal

**Affiliations:** Department of Biology, Brandeis University, 415 South St, Waltham, MA 02454, USA

**Keywords:** *Drosophila*, CRISPR, Golic+, R recombinase/Rippase, Gene targeting, Vps35, OCRL, Tissue-specific tagging

## Abstract

Fluorescent protein tags have revolutionized cell and developmental biology, and in combination with binary expression systems they enable diverse tissue-specific studies of protein function. However these binary expression systems often do not recapitulate endogenous protein expression levels, localization, binding partners and/or developmental windows of gene expression. To address these limitations, we have developed a method called T-STEP (tissue-specific tagging of endogenous proteins) that allows endogenous loci to be tagged in a tissue specific manner. T-STEP uses a combination of efficient CRISPR/Cas9-enhanced gene targeting and tissue-specific recombinase-mediated tag swapping to temporally and spatially label endogenous proteins. We have employed this method to GFP tag OCRL (a phosphoinositide-5-phosphatase in the endocytic pathway) and Vps35 (a Parkinson's disease-implicated component of the endosomal retromer complex) in diverse *Drosophila* tissues including neurons, glia, muscles and hemocytes. Selective tagging of endogenous proteins allows, for the first time, cell type-specific live imaging and proteomics in complex tissues.

## Abstract (precis)

**Summary:** Here we describe an easily applicable method that allows C-terminal protein tagging at endogenous loci in a tissue- or cell type-specific manner in *Drosophila melanogaster*.

## INTRODUCTION

Cellular and developmental biology has been transformed by the application of fluorescent tags, enabling the localization and live imaging of specific proteins and biochemical isolation of their binding partners, among a large number of diverse applications. In *Drosophila melanogaster*, the introduction of the first binary UAS-Gal4 system in 1993 (Brand and Perrimon, 1993) allowed for the tissue specific expression and analysis of proteins of interest, including fluorescently tagged proteins. Even though the UAS-Gal4 and other binary expression systems are indispensable in any *Drosophila* laboratory, in some experimental contexts they do not recapitulate endogenous protein levels and regulatory elements. In these scenarios, toxicity and non-physiologically relevant protein localization or activity can often arise, either from artificially high protein expression levels or from ectopic expression in tissues where the gene of interest does not naturally function.

**Abbreviations:**

T-STEP: tissue-specific tagging of endogenous proteins
Golic+: gene targeting during oogenesis with lethality inhibitor and CRISPR/Cas (Golic+)
R: Rippase recombinase
RRS: Rippase recognition sequence
CNS: central nervous system
NMJ: neuromuscular junction
PTU: phenylthiourea
FLP: Flippase
FRT: Flippase recognition target

Given our interest in identifying the native localization pattern and binding partners of endocytic proteins throughout different stages of development, we sought to eliminate these common shortcomings while preserving the tissue-specificity of the binary expression systems. We designed our method to be economical and easily adopted by any laboratory. By combining the highly efficient lethality selection based gene targeting approach (Chen et al., 2015) with a recently introduced recombinase R (which we refer to as Rippase) from the yeast *Zygosaccharomyces rouxii* (Nern et al., 2011) here we demonstrate the efficiency and effectiveness of the T-STEP method to tissue-specifically label any protein, allowing for cell type-specific imaging and biochemical analysis at endogenous levels.

## RESULTS AND DISCUSSION

### Rationale for T-STEP

**Fig. 1.**
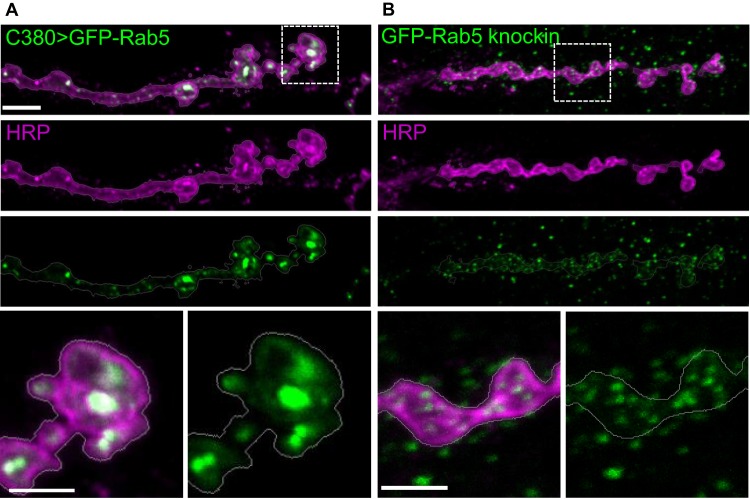
**Overexpression of the endosomal marker GFP-Rab5 changes its localization and distribution pattern.** (A) C380-Gal4-driven UAS-GFP-Rab5 localizes to large punctate compartments at neuronal termini (outlined by HRP staining) that innervate larval muscles, and appear quite different from (B) endogenously expressed GFP-Rab5 (Fabrowski et al., 2013) compartments, which are smaller in size and fairly uniformly distributed. In GFP knock-in animals, GFP-Rab5 is also visible in the postsynaptic muscle tissue, reflecting its endogenous expression pattern. Muscle 6/7 NMJ is shown from segment A3. Scale bars are 5 µm for top panels and 2.5 µm for magnified bottom panels.

Binary expression systems in *Drosophila*, such as the UAS-Gal4, LexAop2-LexA and QUAS-QF2, offer tissue-selective visualization and manipulation of genes of interest. However, these methods do not faithfully recapitulate endogenous protein expression levels and/or localization. An example of such an effect and the dramatic improvement that can be achieved by genomic tagging is shown in Fig. 1 at the *Drosophila* third instar larval neuromuscular junction (NMJ). In this example, the endogenously GFP-tagged Rab5 protein (Fabrowski et al., 2013), a marker for early endosomes, exhibits very different localization from a UAS-GFP-Rab5 transgene expressed with the neuronal C380-Gal4 driver. While the endogenous GFP-Rab5 localizes to small, fairly uniform puncta, in both the motor neuron and in surrounding muscle tissue, neuronally overexpressed GFP-Rab5 is concentrated in enlarged compartments. Thus, overexpression of Rab5 dramatically changes its localization.

An obvious avenue to eliminate overexpression artifacts is to tag endogenous proteins. Numerous approaches for endogenous gene tagging have been developed, including MiMIC (Nagarkar-Jaiswal et al., 2015), FlyTrap insertions (Kelso et al., 2004), homologous recombination mediated genome engineering (Maggert et al., 2008) or CRISPR based genome editing (Gratz et al., 2014; Port et al., 2014). While endogenous gene tagging resolves overexpression issues, these approaches do not enable tissue-specific labeling, which is a prerequisite for imaging and biochemical isolations from tissues or cell types of interest. Chen et al. (2014) developed Synaptic Tagging with Recombination (STaR) to overcome this obstacle, by engineering BAC clones that tag specific pre- and postsynaptic proteins in a tissue-specific manner using recombinases Flippase and Rippase (Chen et al., 2014). However, this powerful method requires laborious BAC engineering for each gene, and further does not replace the endogenous allele.

**Fig. 2.**
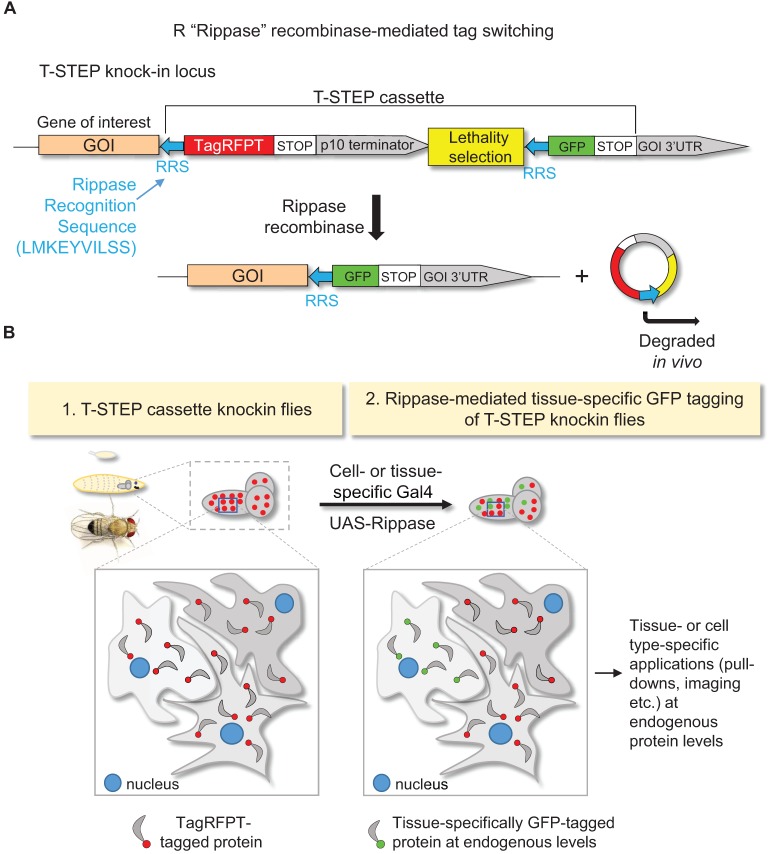
**Conceptual design of the tissue-specific tagging of endogenous proteins (T-STEP) method.** (A) Schematic outline of the genomic locus after T-STEP cassette knock-in using gene targeting. Translation of the targeted protein yields a TagRFPT-tagged protein with the translated Rippase recognition sequence, RRS, serving as a short peptide linker. Upon tissue-specific expression of Rippase the DNA sequence between the tandem RRS sequences is excised and degraded, rendering the protein of interest GFP-tagged and under fully endogenous 5′ and 3′ regulatory elements. The lethality selection module (in yellow) is used only during the gene-targeting step. (B) Before Rippase expression, the targeted protein is TagRFPT-tagged in all cells where it is naturally expressed. Tissue- or cell-type specific protein labeling is achieved by Rippase expression in the desired cell- or tissue type using the UAS-Gal4 system.

We addressed these limitations by designing a CRISPR/Cas9-based gene targeting cassette, T-STEP (tissue-specific tagging of endogenous proteins), comprised of two key components, (a) tandem Rippase specific recognition sequences (RRS) in frame with the targeted protein, which allows tissue-specific tag switching and (b) a lethality selection cassette for very high efficiency gene targeting (Chen et al., 2015) (Fig. 2 and Fig. S1). Recombinase R, or Rippase, was identified in yeast *Zygosaccharomyces rouxii,* and it is one of four novel site-specific recombinases recently adopted in flies (Nern et al., 2011). Like other recombinases, Rippase mediates extremely efficient DNA exchange between two Rippase specific recognition sequences (RRS), and is fully compatible with other existing genetic tools such as FLP/FRT. Most relevant for the T-STEP method, the recognition target sequence of Rippase (RRS, blue arrows in Fig. 2 and Fig. S1A) can be translated without stop codons, and when in frame with the tagged protein, it serves as a short peptide linker between the C-terminus of the targeted protein and the TagRFPT or GFP tag (Fig. 2, Fig. S1B,C). Another crucial component of our approach is the extremely efficient lethality selection cassette adapted from Golic+ (Chen et al., 2015), without which T-STEP would not be easily accessible for many fly labs. The Golic+ method relies on an artificial miRNA gene which suppresses the LexA-driven expression of lethal Rac1^V12^ mutation (riTS-Rac1^V12^). However, the miRNA and its target riTS-Rac1^V12^ are designed to only come in contact (and therefore suppress the Rac1^V12^ inducible lethality) when successful homologous recombination-mediated gene editing has occurred. All other events, such as partial or unsuccessful donor excision and non-specific targeting, result in Rac1^V12^-induced lethality (for detailed information on the design features of lethality selection see Chen et al., 2015). By comparison, existing gene targeting methods where non-specific events are viable (Gratz et al., 2015, 2014; Zhou et al., 2012) require laborious molecular or visual screening of very large numbers of candidate lines. Furthermore, since the choice of location of the CRISPR-mediated dsDNA break is highly restricted in T-STEP by the desired site of tag insertion (Fig. S1B), the number of available gRNA target sequences is limited, which may necessitate the use of gRNA sequences with low efficiencies. Lethality selection easily compensates for potentially low-efficiency gRNAs by simply scaling up the number of crosses without any extra effort at injection or screening. Thus lethality selection allows any laboratory without access to large-scale embryo injection facilities to target any gene with the T-STEP cassette in a virtually fail-proof manner, with unprecedented ease and speed (see Chen et al., 2015). We have also generated a 3xP3-dsRed marked version of the T-STEP vector for labs preferring injection based gene targeting with visual screening for targeted events (Gratz et al., 2014) (Fig. S4C).

We tested our approach by T-STEP tagging wild-type Vps35 (2nd chromosome) (see Fig. S1B for the details of the targeting steps) and OCRL (X chromosome). For Vps35, we made a second targeting construct that also carried the conserved Parkinson's disease-linked D628N linked mutation (Zimprich et al., 2011) in the 5′ homology arm. The donor vectors were inserted into the appropriate attP docking site via standard transgenesis. Following a simple crossing scheme with published stocks (Chen et al., 2015) (Fig. S5) we obtained targeted events for all three constructs with very high efficiency (see Table S1), which were further confirmed by western blotting and PCR (Fig. S2).

### *In vivo* characterization of T-STEP knock-ins

Imaging fixed third instar larval tissues demonstrated the subcellular localization of endogenous OCRL-TagRFPT and Vps35-TagRFPT protein, respectively (Fig. S3) as well as their subcellular dynamics upon live imaging (see Movies 1-3). In hemocytes, OCRL-TagRFPT localized to small, fairly uniformly distributed structures throughout the cytoplasm, likely of endocytic origin, as well as in the nucleus (Fig. S3A). Vps35-TagRFPT was expressed at higher levels than OCRL-TagRFPT, and was the focus of our remaining experiments. Vps35-TagRFPT was readily visible in most tissues, including the nervous system, epithelia, muscles, and hemocytes, where Vps35 has previously been shown to function (Dong et al., 2013; Korolchuk et al., 2007). Live imaging of Vps35-TagRFPT in hemocytes revealed its dynamic association relative to Rab5- or Rab11-positive endosomes (Movies 1 and 2). In fixed larval muscle cells Vps35-TagRFPT was found in small, distributed puncta and in larger perinuclear structures (Fig. S3B,C). Thus, the T-STEP cassette efficiently reports the expected localization of targeted endogenous proteins.

### Tissue-specific Rippase-mediated GFP tagging of T-STEP knock-ins

**Fig. 3.**
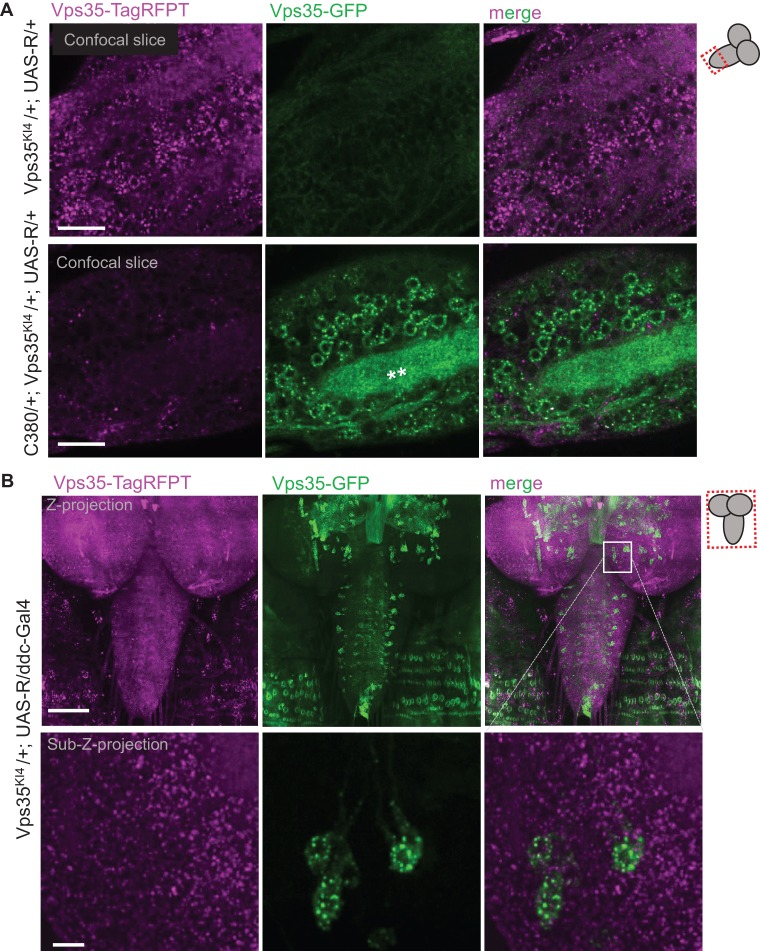
**Tissue-specific tagging with T-STEP.** (A) Motorneuron-specific GFP-tagging of endogenous Vps35. In comparison with control animals (top panel) that do not express a Gal4 driver, Vps35^KI4^ larval brains that express UAS-Rippase driven by C380-Gal4 (which drives in many glutamatergic motor neurons) reveal the appearance of punctate Vps35-GFP signal in neuronal cell bodies as well as the neuropil (white stars in bottom panel). Identical acquisition settings were used for both genotypes. Single confocal sections are shown from the area of the ventral ganglion outlined in the cartoon scheme on the right. Scale bars are 30 µm. (B) Dopaminergic and serotonergic neuron-specific GFP-tagging of endogenous Vps35. GFP-tagging Vps35 in a smaller set of serotonergic and dopaminergic neurons using the ddc-Gal4 driver highlights the strengths of the T-STEP method not only for imaging at unprecedented detail at endogenous levels, but also for opening the possibilities for neuron-type specific pull-downs of binding partners of proteins of interest. Maximum intensity *Z*-projection is shown (175 µm stack) for top panel and 10 µm sub-stack for bottom panel. Green signal outside of the nervous system reflects autofluorescence of the body wall denticles. Scale bars are: top panels 100 µm, bottom panels 10 µm. Endogenous GFP and TagRFPT signals were acquired without antibody staining.

**Fig. 4.**
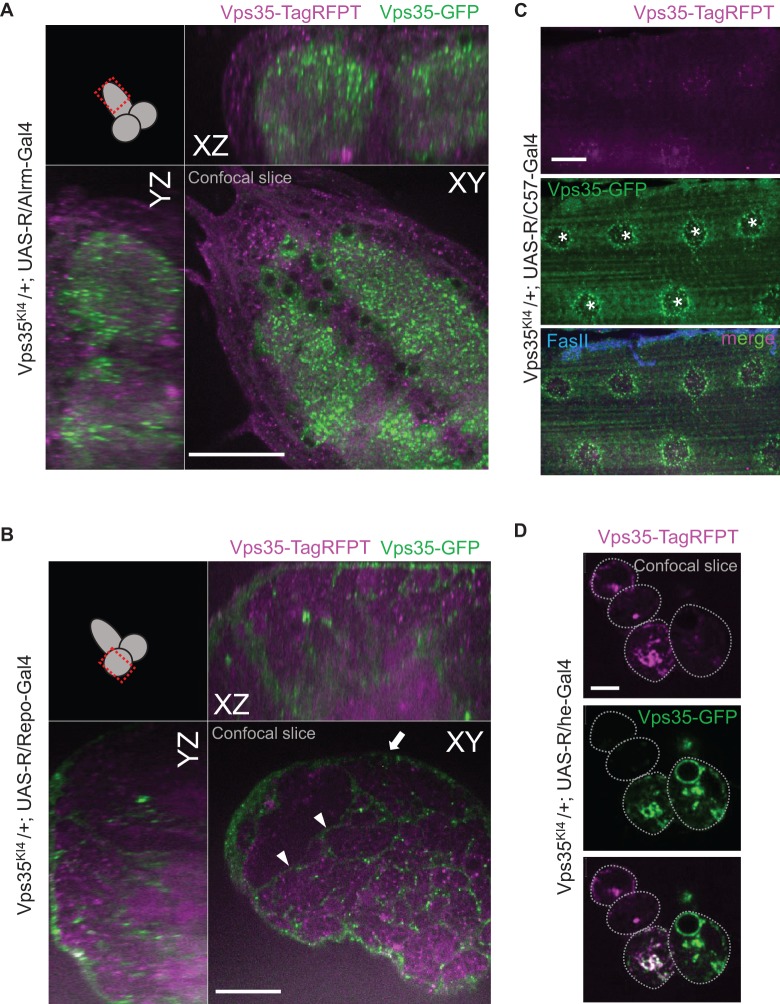
**Vps35 tagging in astrocytes, pan-glial populations, larval muscles and larval hemocytes.** (A) Astrocyte-specific Vps35 GFP tagging. *YZ* and *XZ* single confocal planes demonstrating the subcellular distribution of Vps35-GFP throughout the larval astrocyte population, including their processes infiltrating the neuropil. Scale bar is 40 µm. (B) Repo-Gal4-mediated pan-glial tag conversion reveals surface glial (arrow) and cortex glial (arrowheads) expression of Vps35 in a single confocal plane through the lobe of the larval CNS. Scale bar is 40 µm. (C) Larval muscle-specific Vps35 GFP tagging. Maximum intensity projection of a confocal *Z*-stack through a larval muscle cell M6 (which are multi-nucleate, marked by stars) that expresses Rippase with the muscle driver C57-Gal4. Vps35-GFP is present most prominently around the muscle nuclei though it can be seen in small punctate structures throughout the muscle cell. Synaptic termini were stained with FasII. Scale bar is 25 µm. (D) Hemocyte-specific Vps35 GFP tagging. Single confocal slice through a cluster of four third instar larval hemocytes from Vps35^KI4^ knock-in animals that express Rippase with the hemese-Gal4 driver in the majority of hemocytes (Zettervall et al., 2004). The top two cells express Vps35-TagRFPT almost exclusively, with only very low level of Vps35-GFP signal detectable, indicating that Vps35-GFP translation has not progressed long enough for the signal to become readily visible. The other hemocytes accumulated varying amounts of Vps35-GFP depending on their cellular birth date and the relative timing of R-mediated conversion. Scale bar is 5 µm.

To test whether tissue-specific expression of the Rippase could lead to the conversion of Vps35-TagRFPT to Vps35-GFP, we employed a range of tissue-specific Gal4 drivers. In all tissues tested we observed the appearance of Vps35-GFP (Figs 3, 4), in accord with the very high efficiency of Rippase mediated events reported previously (>96%; Nern et al., 2011). In a population enriched for glutamatergic motor neurons (C380-Gal4), Vps35-GFP was detected in neuronal cell bodies as well as the neuropil (Fig. 3A). When we expressed Rippase using ddc-Gal4 [which expresses Gal4 in a subset of dopaminergic and serotonergic neurons (Li et al., 2000)], the Vps35-GFP signal revealed in unprecedented detail the subcellular localization of Vps35 in a tissue type implicated in Parkinson's disease (Fig. 3B). When tagged in astrocytes, Vps35-GFP localized to astrocyte cell bodies as well as to processes infiltrating the neuropil (Fig. 4A). Pan-glial tagging using Repo-Gal4 revealed that Vps35 is expressed in a number of diverse glia types (Fig. 4B). In larval muscles, Vps35 was most prominent around the muscle nuclei (Fig. 4C). In hemocytes, Vps35-GFP was readily observed in the same pattern as Vps35-TagRFPT (Fig. 4D). In this tissue type we noted some variability in the ratio of Vps35-TagRFPT to Vps35-GFP (Fig. 4D) likely reflecting a combination of factors ranging from Vps35 protein half-life, strength of the Gal4 driver, tissue or cell-type specific protein levels, and the timing of the Rippase-mediated event relative to cell division. These variables of the T-STEP system could potentially be exploited to assess the half-life of proteins before and after Rippase-mediated conversion in specific tissue-types or during specific developmental windows.

One potential caveat of any protein tagging system is that the tag could interfere with protein function, localization or degradation. The Vps35 and OCRL homozygous T-STEP knock-in flies are fertile and viable (compared to null mutants, which are larval lethal (Korolchuk et al., 2007; our unpublished results) and the Vps35^KI^ allele complements a small chromosomal deficiency lacking Vps35 (our unpublished results). The TagRFPT-tagged version of the knock-ins carries the p10 terminator from *Autographa californica* nucleopolyhedrovirus, while the GFP-tagged rip-out allele is under fully endogenous regulatory elements. Thus, it is possible that their regulation might be different. However, the identical localization pattern of Vps35 (Figs 3, 4) and OCRL (data not shown) before and after tag conversion argues that in the case of these two proteins, the p10 3′UTR does not negatively interfere with expression patterns or localization. Although p10 was initially chosen to minimize the presence of repetitive regions in the donor vector, it should also be possible to use endogenous 3′ regulatory elements for both TagRFPT and GFP tagged versions of the targeted proteins.

One of the inherent drawbacks of our and other genomic tagging approaches is that they may be of limited use for proteins with very low expression levels. We have prepared T-STEP cassettes with alternative tags, such as SNAPf, which may offer further flexibility and sensitivity for certain applications (Kohl et al., 2014). In addition, T-STEP could be used to simultaneously label both an mRNA and its cognate protein in a tissue-specific manner, by incorporating RNA-tagging recognition sequences in the 3′UTR of the targeting cassette. This would allow the method to be extended for the tissue-specific identification of protein and/or mRNA binding partners at endogenous levels. Furthermore, T-STEP offers unique opportunities to facilitate the mechanistic understanding of diverse tissue-specific diseases. For example, in many neurological diseases select neuronal populations are predominantly affected (e.g. motor-neurons in amyotrophic lateral sclerosis, or dopaminergic subpopulations in Parkinson's disease), even though every cell of the organism carries the causative mutation. By using T-STEP and taking advantage of existing and rapidly expanding (Diao et al., 2015) tissue-specific drivers, one can selectively visualize, analyze or isolate protein or RNA from the affected tissues of wild type or mutant animals at native levels, a possibility that has not been feasible until now. In summary, the T-STEP approach affords a simple and robust method to tissue-specifically label proteins at their C-termini at endogenous levels, and with comparable cloning effort that is required for routine binary expression constructs (UAS/LexAop/QUAS).

## MATERIALS AND METHODS

### DNA constructs

Standard molecular biology techniques and Gibson cloning were used to generate all plasmids and intermediates. The pT-STEP donor plasmids for C-terminally tagged GFP-swappable TagRFPT fusions incorporate the recently published lethality selection [from vector pTL2 (Chen et al., 2015)] with the following modifications: The TagRFPT coding region was amplified from TagRFPT-EEA1, (Addgene plasmid #42635, from Silvia Corvera, UMASS Medical Schoool, Worcester, MA, USA) with primers incorporating the Rippase recognition sequence RRS (in −1 frame relative to the directionality of RRS so that no stop codons are present: TTGATGAAAGAATACGTTATTCTTTCATCAA) in frame at the 5′ of TagRFPT, leading to a short linker peptide when translated (LMKEYVILSS-S-TagRFPT). The 3′-UTR from the *Autographa californica* nucleopolyhedrovirus (AcNPV) p10 gene was amplified from pJFRC81-10XUAS-IVS-Syn21-GFP-p10, (Addgene plasmid #36432, from Gerald Rubin, Janelia Farms, Ashburn, VA, USA), chosen for its efficiency in female germline cells (Pfeiffer et al., 2012). The LoxP-DsRed-LoxP cassette was obtained from pDsRed-attP (Addgene plasmid #51019, from Melissa Harrison, Kate O'Connor-Giles and Jill Wildonger, University of Wisconsin, Madison, WI, USA). The PreScission Protease (PSP) recognition sequence (TTGGAGGTCCTGTTCCAGGGCCCC/LEVLFQ^GP) followed by a short linker (GSGSGS) and GFP were synthesized as a gene block (IDT DNA, Iowa, USA). The second RRS recognition sequence (−1 frame) was included in the 5′ primer used to amplify the PSP-GFP gene block.

The BspQI sites within pTL2 were changed to BsmBI (TagRFPT contains a BspQI site). Components of the swappable-to-GFP cassette were first assembled in a PCR4-TOPO vector yielding the intermediate vector. Using this intermediate as a template, the RRS-TagRFPT-p10-LoxP region was PCR amplified and inserted between the NsiI and SpeI of pTL2, thereby removing the original I-CreI and attPX sites. The resulting plasmid was then digested with PacI and BamHI and, using Gibson cloning, the RRS-PSP-GFP was inserted yielding the final pT-STEP vector, which has been deposited at Addgene. The pT-STEP-SNAPf vector is identical to pT-STEP except that a SNAPf tag (New England Biolabs) instead of the GFP tag is introduced after the Rippase reaction (see also Fig. S4). pDsRed-TSTEPv2, assembled in a pBlueScript backbone (Fig. S4), is suitable for embryo injection mediated gene targeting and contains the TagRFPT to GFP convertible cassette in addition to a 3xP3 promoter-driven loxP flanked DsRed selection marker from the Addgene #51019 plasmid. All vector details are available on request and from Addgene (Addgene plasmid numbers pTSTEP #72334; pTSTEP-SNAPf #72335; pDsRed-TSTEP_v2 #72336).

Oligos corresponding to CRISPR Cas9 target sites (Vps35 [t]agcccagcgcacccactt and OCRL [c]cgcagctgtgccgccgaat) and containing 4 extra base pairs for BsmBI compatibility (see Fig. S5) were annealed and ligated the BsmBI site of pT-STEP (bases in [brackets] were changed to the obligatory G for the dU6.3 promoter). For introducing the Parkinson's disease specific human Vps35^D620N^ mutation (corresponding to D628N in *Drosophila melanogaster* Vps35), the 5′ arm of wild type Vps35 was subcloned into pJet1.2 vector and Gibson cloning was used to introduce the specific mutation. Three targeting vectors were made (OCRL, Vps35 and Vps35^D628N^). The 5′ homology arms of wild type or D628N mutant Vps35 (2R:22185904..22189272) or OCRL (X:1924260..1927163) were inserted at the StuI site of pT-STEP, and their respective 3′ arms (2R:22189273..22192812) and (X: 1927164..1930079) in the PmeI site (numbers reflect the DGRC r6.05 database). The Vps35 3′ homology arm was modified to abolish the PAM region of the chosen target sequence, while the OCRL targeting construct did not carry resistance to Cas9.

### Fly strains

Flies carrying the UAS-Rippase followed by a short PEST sequence (aa 422–461 of the mouse ornithine decarboxylase gene) to decrease its half-life and potential toxicity (pJFRC165-20XUAS-IVS-R::PEST in attP2) were from Gerald Rubin (Janelia Farms, Ashburn, VA, USA); Alrm-Gal4 was from Marc Freeman (UMASS Medical School, Worcester, MA, USA), C380-Gal4 and C57-Gal4 were from Vivian Budnik (UMASS Medical School, Worcester, MA, USA), UAS-GFP-Rab5 from Marcos Gonzalez-Gaitan (University of Geneva, Switzerland) and GFP-Rab5 knock-in flies (Fabrowski et al., 2013) from Stefano De Renzis [European Molecular Biology Laboratory (EMBL) Heidelberg, Germany]. YFP-HA-Rab11 knock-in flies (Dunst et al., 2015) were from Marko Brankatschk (Max Planck Institute of Molecular Cell Biology and Genetics, Germany), Hemese-Gal4 (#8699), Repo-Gal4 (#7415), ddc-Gal4 (#7009) and w; Df(exel)6078/CyO (#7558) were from the Bloomington Drosophila Stock Center.

### Gene targeting with lethality selection (Golic+)

The donor plasmid for OCRL was injected to attP40 docking site and for Vps35 to the VK00027 docking site by Rainbowgene Inc, CA, USA. All Golic+ strains were from Hui-Min Chen and Tzumin Lee (Janelia Farms, Ashburn, VA, USA) (Chen et al., 2015), and the bam898-Cas9-2A-FLP-2A-ISceI stocks were generously shared before publication (see also Fig. S5 for detailed workflow). Transformants were screened for by crossing to the Pin/CyO; GMR3-LexA in attP2 and GMR3-LexA in attP40; TM3, Sb/TM6B, Tb, respectively, using the reduced eye size phenotype. The Vps35 donor lines (Vps35-790.25.F1 in VK00027and Vps35^D628N^-795.46.M4 in VK00027) were crossed to bam898-Cas9-2A-FLP-2A-I-SceI in su(Hw)attP8/FM6c; Pin/CyO; LexAop2-5xriTS-Rac1^V12^ in VK00027/TM3, Sb strain while the OCRL donor line (OCRL-793.R39.1 in attP40) to the LexAop2-5xriTS-Rac1^V12^ in attP40/CyO; bam898-Cas9-2A-FLP-2A-I-SceI in attP2/TM3, Sb. Lethality selection was then performed using the Pin/CyO; nSyb-LexA in VK00027 for Vps35 and FM7a; nSyb-LexA in attP16 for OCRL. Candidates were mapped using the Pin/CyO; GMR>riTS-Rac1^V12^ line.

### Antibodies

FasII 1D4 (1:10), LamDm0 (1:500) and LamC (1:30) monoclonal antibodies were from Developmental Studies Hybridoma Bank (University of Iowa). Anti-TagRFP antibody (1:1500 for western blotting) was from Evrogen (AB234). α-HRP antibodies and secondary antibodies for imaging were conjugated to Alexa Fluor 488 or Alexa Fluor 647 (Jackson Immunoresearch, West Grove, PA, USA). Anti-rabbit DyLight 680 antibody (1:5000) was from Rockland, PA.

### Hemocyte preparation and imaging

Hemolymph from third instar larvae was bled onto coverslips into a drop of phosphate buffered saline (PBS) containing 20 µM phenylthiourea (PTU) and allowed to adhere for 5 min at room temperature. Hemocytes were either imaged live by mounting the coverslip on a glass slide with narrow spacer, or fixed in 4% paraformaldehyde on the coverslips in PBS for 10 min and then stained with the indicated antibodies.

### Western blotting

Hemolymph from ten third instar larvae was collected into 10 µl of 20 µM PTU in PBS as illustrated in https://www.youtube.com/watch?v=im78OIBKlPA. An equal volume of 2× sample buffer (Bio-Rad) was added and samples were heated for 5 min at 95°C. 15 µl (3.8 larvae) were loaded onto 7.5% polyacrylamide gels (Bio-Rad). Proteins were transferred to nitrocellulose membrane and blocked in blocking buffer for fluorescent western blotting (Rockland, PA, USA). The membrane was probed with rabbit anti-TagRFP antibodies followed by anti-rabbit DyLight 680, and visualized on Licor Odyssey Scanner (Lincoln, NE, USA).

### Larval dissections and imaging

Third instar larvae were dissected in HL3.1 (Feng et al., 2004) and fixed in 4% paraformaldehyde in HL3.1 for 10 min at room temperature, then rinsed and stained with appropriate antibodies in PBS containing 0.2% (v/v) Triton X-100. Larvae were mounted in Vectashield (Vector Labs, Burlingame, CA, USA). Spinning-disk confocal *Z*-stacks (0.3 μm or 1 µm) were collected at room temperature on an Andor spinning-disk confocal system consisting of a Nikon Ni-E upright microscope equipped with 40× [numerical aperture (NA) 1.3], 60× (NA 1.4) and 100× (NA 1.45) oil immersion objectives, or a 60× (NA 1.0) water immersion objective, a Yokogawa CSU-W1 spinning-disk head, and an Andor iXon 897U electron-multiplying charge-coupled device camera (Andor, Belfast, Northern Ireland). Images were collected using Nikon Elements AR software and processed using Volocity software (Improvision, PerkinElmer, Waltham, MA, USA).
